# Use of a Novel Network-Based Linchpin Score to Characterize Accessibility to the Oncology Physician Workforce in the United States

**DOI:** 10.1001/jamanetworkopen.2022.45995

**Published:** 2022-12-16

**Authors:** Erika L. Moen, Gabriel A. Brooks, A. James O’Malley, Andrew Schaefer, Heather A. Carlos, Tracy Onega

**Affiliations:** 1 Department of Biomedical Data Science, Geisel School of Medicine at Dartmouth, Lebanon, New Hampshire; 2 The Dartmouth Institute for Health Policy and Clinical Practice, Lebanon, New Hampshire; 3 Dartmouth Cancer Center, Dartmouth-Hitchcock Medical Center, Lebanon, New Hampshire; 4 Department of Medicine, Geisel School of Medicine at Dartmouth, Lebanon, New Hampshire; 5 Huntsman Cancer Institute, University of Utah, Salt Lake City; 6 Department of Population Health Science, University of Utah, Salt Lake City

## Abstract

**Importance:**

Physician headcounts provide useful information about the cancer care delivery workforce; however, efforts to track the oncology workforce would benefit from new measures that capture how essential a physician is for meeting the multidisciplinary cancer care needs of the region. Physicians are considered linchpins when fewer of their peers are connected to other physicians of the same specialty as the focal physician. Because they are locally unique for their specialty, these physicians’ networks may be particularly vulnerable to their removal from the network (eg, through relocation or retirement).

**Objective:**

To examine a novel network-based physician linchpin score within nationwide cancer patient-sharing networks and explore variation in network vulnerability across hospital referral regions (HRRs).

**Design, Setting, and Participants:**

This cross-sectional study analyzed fee-for-service Medicare claims and included Medicare beneficiaries with an incident diagnosis of breast, colorectal, or lung cancer from 2016 to 2018 and their treating physicians. Data were analyzed from March 2022 to October 2022.

**Exposures:**

Physician characteristics assessed were specialty, rurality, and Census region. HRR variables assessed include sociodemographic and socioeconomic characteristics and use of cancer services.

**Main Outcomes and Measures:**

Oncologist linchpin score, which examined the extent to which a physician’s peers were connected to other physicians of the same specialty as the focal physician. Network vulnerability, which distinguished HRRs with more linchpin oncologists than expected based on oncologist density. χ^2^ and Fisher exact tests were used to examine relationships between oncologist characteristics and linchpin score. Spearman rank correlation coefficient (ρ) was used to measure the strength and direction of relationships between HRR network vulnerability, oncologist density, population sociodemographic and socioeconomic characteristics, and cancer service use.

**Results:**

The study cohort comprised 308 714 patients with breast, colorectal, or lung cancer. The study cohort of 308 714 patients included 161 206 (52.2%) patients with breast cancer, 76 604 (24.8%) patients with colorectal cancer, and 70 904 (23.0%) patients with lung cancer. In our sample, 272 425 patients (88%) were White, and 238 603 patients (77%) lived in metropolitan areas. The cancer patient-sharing network included 7221 medical oncologists and 3573 radiation oncologists. HRRs with more vulnerable networks for medical oncology had a higher percentage of beneficiaries eligible for Medicaid (ρ, 0.19; 95% CI, 0.08 to 0.29). HRRs with more vulnerable networks for radiation oncology had a higher percentage of beneficiaries living in poverty (ρ, 0.17; 95% CI, 0.06 to 0.27), and a higher percentage of beneficiaries eligible for Medicaid (ρ, 0.21; 95% CI, 0.09 to 0.31), and lower rates of cohort patients receiving radiation therapy (ρ, –0.18; 95% CI, –0.28 to –0.06; *P* = .003). The was no association between network vulnerability for medical oncology and percent of cohort patients receiving chemotherapy (ρ, –0.03; 95% CI, –0.15 to 0.08).

**Conclusions and Relevance:**

This study found that patient-sharing network vulnerability was associated with poverty and lower rates of radiation therapy. Health policy strategies for addressing network vulnerability may improve access to interdisciplinary care and reduce treatment disparities.

## Key Points

**Question:**

Can network analysis be used to enhance measurement of the oncology workforce?

**Findings:**

In this cross-sectional study of 308 714 patients, a novel measure of patient-sharing network vulnerability was found to be higher in regions characterized by socioeconomic disadvantage and associated with lower rates of radiation therapy.

**Meaning:**

These findings suggest that efforts to track the oncology workforce and identify populations vulnerable to oncology workforce shortages can benefit from considering the structure of patient-sharing networks.

## Teaser

This cross-sectional applies the linchpin score, a measure of patient-sharing network vulnerability, to oncologists in the US from 2016 to 2018 to assess variation in network vulnerability across hospital referral regions.

## Introduction

The American Society of Clinical Oncology projected oncologist shortages in the United States by 2020, and subsequent forecasts continue to show a demand for cancer services that will exceed supply.^1,2,3^ Stark geographic disparities in the oncology workforce have been noted, particularly in rural areas.^4^ While these reports have provided critical high-level data, they have also raised questions regarding how to best measure the oncology workforce and what constitutes a workforce shortage.^5^

As efforts to track the oncology workforce have advanced, so too has the evidence surrounding the value of multidisciplinary cancer care teams.^6,7,8^ The importance of relationships, particularly those involving multiple specialties and spanning practices, provides a strong rationale for leveraging network analysis to enhance the measurement of the oncology workforce. Herein, we describe a network-based physician linchpin score to capture how vital a physician is for bringing their specialty-specific expertise to their peers.^9^ Taking its name from a small but important metal pin used to prevent a wheel from sliding off its axle, physicians are characterized as a linchpin when fewer of their peers are connected to other physicians of the same specialty as the focal physician. Because they are locally unique for their specialty, we posit that their networks would be particularly vulnerable to their removal from the network (eg, through relocation or retirement). In this scenario, we postulate that new relationships will likely need to be established to maintain delivery of interdisciplinary care, which would take more time and effort than having a relationship already established. The objective of this study was to identify linchpin oncologists and characterize hospital referral region (HRR) network vulnerability for cancer care, making comparisons with the traditional oncologist per capita measure of the oncology workforce, herein referred to as oncologist density.

## Methods

The study was approved by Dartmouth College Committee for the Protection of Human Subjects institutional review board. The data are not publicly available due to the data use agreement with the Centers for Medicare and Medicaid Services. This study followed the Strengthening the Reporting of Observational Studies in Epidemiology (STROBE) reporting guideline.

### Study Cohort

Implementing a published method modified for use with *International Statistical Classification of Diseases, Tenth Revision, Clinical Modification *(*ICD-10-CM*) diagnosis codes, in this cross-sectional study, we identified beneficiaries with a biopsy for breast, colorectal, or lung cancer followed by 2 cancer diagnosis codes in the following 12 months from the 100% sample of fee-for-service Medicare claims from 2015 to 2019 (eTable 1 in Supplement 1).^10^ We excluded patients with a cancer diagnosis code in the 12 months preceding the biopsy to enrich for incident cancer cases. To allow each patient to have 12-month look-forward and look-back windows, our patient cohort included those who received cancer-directed biopsies from 2016 to 2018. Patients were further excluded if they were younger than 65 years or older than 99 years at the time of biopsy, had a missing or non-US ZIP code, or were not continuously enrolled in Medicare Parts A and B until the sooner of death or 12 months following biopsy. Breast, colorectal, and lung cancers were chosen because they are common cancers often requiring multidisciplinary care, so the oncologist linchpin score should be driven primarily by limits to oncologist supply rather than a requirement for highly specialized treatment expertise.

### Study Variables

Physician specialty was identified in the National Plan and Provider Enumeration System file. Physicians were attributed to a practice ZIP code based on the plurality of claims with cohort patients during the study period. Rurality was defined based on the 4-tier categorization of 2010 Rural-Urban Commuting Area codes. HRR sociodemographic characteristics were obtained from the Dartmouth Atlas to examine associations with network vulnerability. Race and ethnicity were obtained from the Research Triangle Institute variable in the Medicare Beneficiary Summary Files. The proportion of cohort patients with at least 1 claim of chemotherapy or radiation therapy was measured using the CPT and HCPCS codes in eTable 2 in Supplement 1. Oncologist density for each HRR was calculated based on the number of oncologists per 10 000 Medicare beneficiaries.

### Network Assembly

Patients with breast, colorectal, and lung cancer formed a combined cancer patient cohort. We identified the physicians who had encounters in the Medicare Part B files with the patients in our cohort in the 3 months prior to and/or 12 months following the cancer-directed biopsy. From these encounters, physician networks were created in which pairs of physicians were connected by edges with values corresponding to the number of shared patients. To reduce network noise and computational burden, physicians who cared for less than 5 patients were excluded, and edges representing less than 3 shared patients were set to null edges.

### Physician Linchpin Score

**Figure 1.**
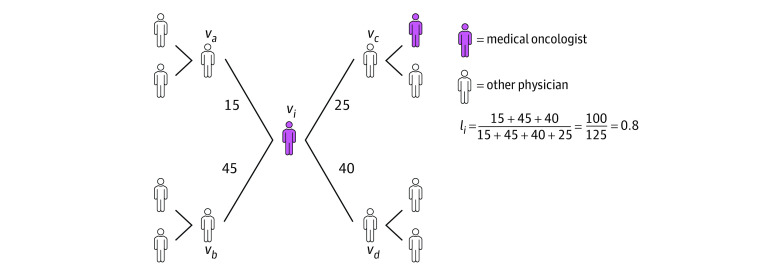
Linchpin Score Calculation Illustration of linchpin score calculation that is found by summing edges with peers who lack ties to another medical oncologist (eg, *v_a_*, *v_b_*, and *v_d_*) and dividing by the sum of all shared ties (eg, *v_a_*, *v_b_*, *v_c_*, and *v_d_*). Numbers adjacent to edge lines represent shared patients.

Linchpin scores for medical and radiation oncologists were calculated based on a nationwide network inclusive of all clinicians who had encounters with cohort patients and met the inclusion criteria. In Figure 1, we illustrate the linchpin score calculation for medical oncologist i(v_i_) by summing edges with peers who lack ties to another medical oncologist (eg, v_a_, v_b_, and v_d_) and dividing by the sum of all shared ties (eg, v_a_, v_b_, v_c_, and v_d_). Medical and radiation oncologists were considered a linchpin if their linchpin score was in the top 15% for their specialty. A range of thresholds was considered in sensitivity analyses (eTable 3 in Supplement 1).

### HRR Network Vulnerability

The physician ZIP code was linked to HRR to facilitate subsequent analyses. Our goal was to construct a measure of HRR network vulnerability that reflected the extent to which the number of linchpin oncologists in a region exceeded the expected number given oncology density. Networks with more linchpins than expected may reflect less redundancy in the way the patient-sharing patterns are formed. This could be due to various reasons, such as low oncologist supply, geographic barriers, and physician or patient preferences, among others. We also wanted our measure to be invariant to the beneficiary population of a region. To estimate the expected number of oncologists or linchpin oncologists based on the number of beneficiaries per HRR, fitted values given the number of beneficiaries in each HRR were generated from a Poisson regression. Then, the observed to expected (O:E) ratios of oncologists and linchpin oncologists per HRR were calculated by dividing the number observed by the expected (ie, fitted) values.

We hypothesized that HRRs with greater O:E ratios of linchpin oncologists adjusting for the oncologist density would be more vulnerable to disruption due to workforce shortages. To calculate a measure of HRR network vulnerability reflective of this, the O:E linchpins per HRR was regressed on O:E oncologists per HRR, and the residuals were calculated to capture how far away each HRR was vertically from the regression line. HRRs above the line have more linchpins than expected given the oncologist density, whereas HRRs below the line have fewer linchpins than expected. Network vulnerability for medical oncology and radiation oncology was calculated separately. We also computed the raw proportion of oncologists who are linchpins and compared this with our vulnerability index. The extent to which the correlation of the 2 measures is less than 1 is a measure of the extent to which the number of beneficiaries in a region drives variation (ie, confounds) the number of linchpin oncologists and the total number of oncologists. An advantage of the proposed vulnerability measure over the proportion of oncologists is that it generalizes automatically to a measure that adjusts for multiple variables, which could be useful in other applications.

### Statistical Analyses

Bivariate analyses were used to explore the relationships between study variables. χ^2^ and Fisher exact tests were used to examine associations between oncologist characteristics and linchpin score. Spearman rank correlation coefficient *(ρ)* was used to measure the strength and direction of correlations between HRR network vulnerability, oncologist density, population sociodemographic and socioeconomic characteristics, and cancer service use. Values range from −1 (perfect inverse relationship) to 1 (perfect positive relationship), with 0 indicating no association. Statistical analyses were performed using R version 4.1.3 (R Project for Statistical Computing) and visualized in ArcGIS version 10.8.2 (Esri). Data were analyzed from March 2022 to October 2022.

## Results

**Figure 2.**
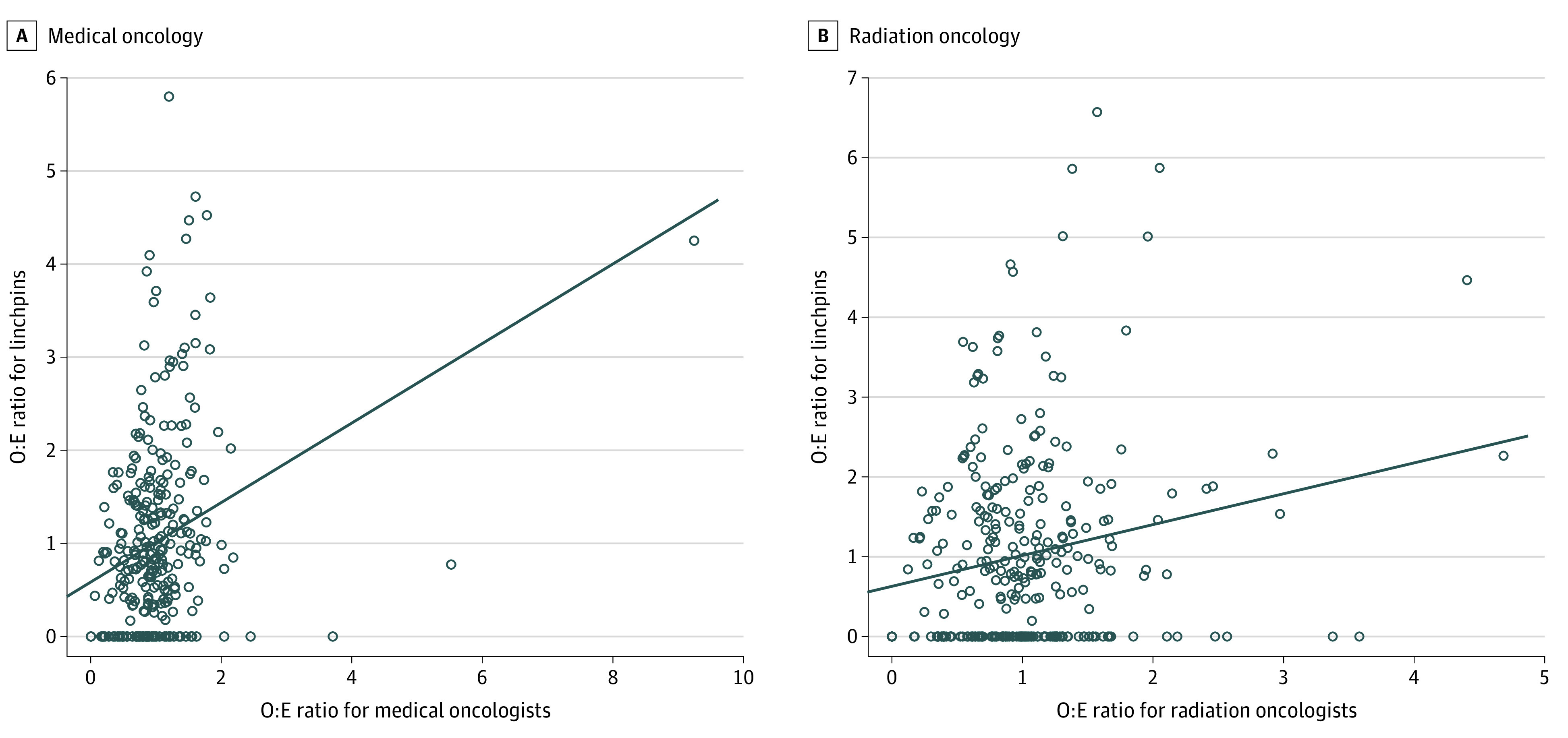
Observed to Expected Linchpin Oncologists Per 10 000 Plot of observed to expected linchpin oncologists per 10 000 Medicare beneficiaries by O:E ratio of oncologists per 10 000 Medicare beneficiaries for (A), medical oncology and (B), radiation oncology. Each point represents a hospital referral region. The vertical distance from each point to the regression line represents hypothesized network vulnerability. O:E indicates observed to expected ratio.

The study cohort of 308 714 patients included 161 206 (52.2%) patients with breast cancer, 76 604 (24.8%) patients with colorectal cancer, and 70 904 (23.0%) patients with lung cancer (eFigure 1 in Supplement 1). In our sample, 272 425 patients (88%) were White, and 238 603 patients (77%) lived in metropolitan areas (eTable 4 in Supplement 1). The cancer patient-sharing network included 140 140 clinicians of which 7221 (5%) were medical oncologists, 3573 (3%) were radiation oncologists, 18 528 (13%) were surgeons, 42 578 (30%) were general practitioners, and the remainder were other specialists. Characteristics of linchpin medical oncologists and radiation oncologists are described in Table 1. Compared with their metropolitan counterparts, medical oncologists were more likely to be a linchpin when they practiced in micropolitan ZIP codes, and radiation oncologists were more likely to be a linchpin when they practiced in micropolitan or small-town ZIP codes (Table 1). The O:E ratios of linchpin oncologists per HRR are plotted against the O:E ratios of oncologists per HRR in Figure 2. The vertical distance between the point and the regression line represents hypothesized HRR network vulnerability.

**Table 1.** Characteristics of Oncologists in the Nationwide Cancer Patient-Sharing Network^a^

Characteristic	Medical oncology			Radiation oncology		
	Linchpin (n = 1086)	Not a linchpin (n = 6135)	*P* value	Linchpin (n = 538)	Not a linchpin (n = 3035)	*P* value
Rurality						
Metropolitan	939 (14.6)	5512 (85.4)	.006	448 (13.8)	2788 (86.2)	<.001
Micropolitan	130 (19.8)	528 (80.2)		80 (27.6)	210 (72.4)	
Small town	14 (13.1)	79 (84.9)		9 (28.1)	23 (71.9)	
Isolated	3 (15.8)	16 (84.2)		1 (6.7)	14 (93.3)	
Census region						
Northeast	276 (18.4)	1223 (81.6)	<.001	143 (20.5)	555 (79.5)	<.001
Midwest	257 (14.9)	1467 (85.1)		99 (11.1)	789 (88.9)	
South	330 (12.2)	2369 (87.8)		183 (14.4)	1084 (85.6)	
West	223 (17.2)	1076 (82.8)		113 (15.3)	627 (84.7)	

^a^ *P* values calculated using Fisher exact test or Pearson χ^2^ tests. Percentages are by row.

**Figure 3.**
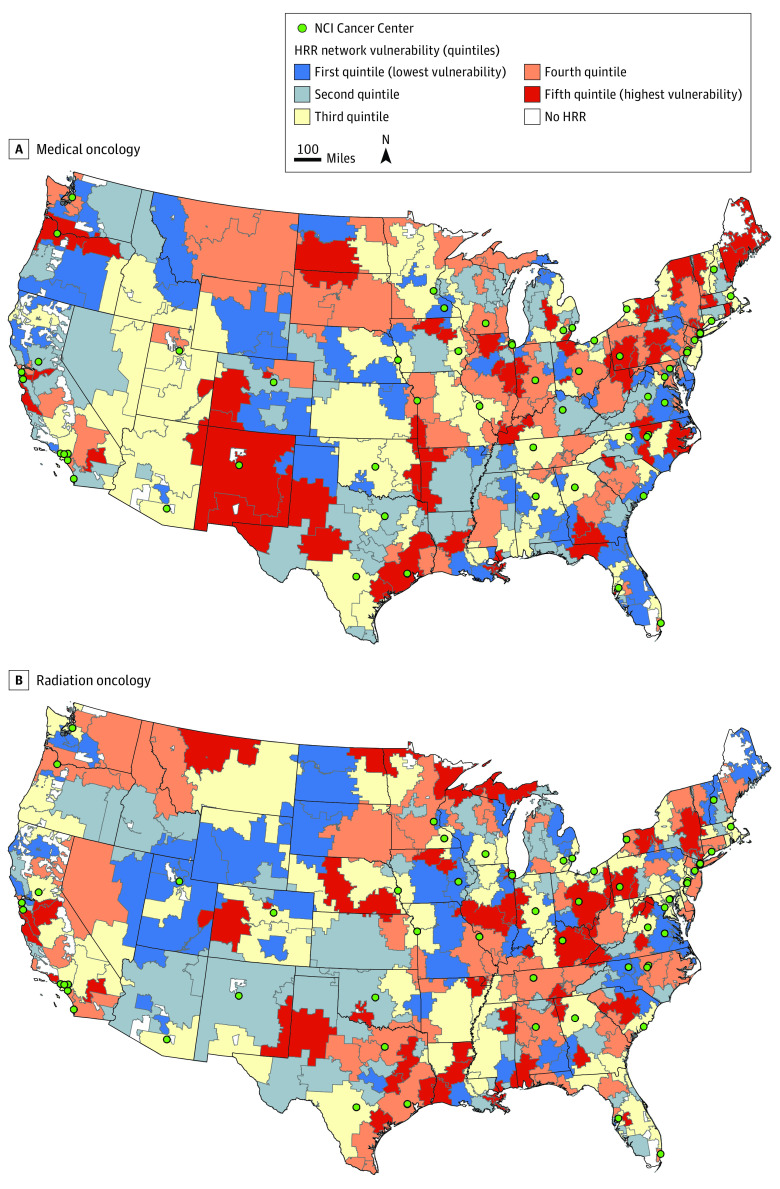
Geographic Variation in HRR Network Vulnerability Geographic variation in the HRR network vulnerability for (A), medical oncology and (B), radiation oncology. HRR indicates hospital referral regions; NCI, National Cancer Institute.

We observed substantial geographic variation in HRR network vulnerability (Figure 3). Network vulnerability was strongly and positively associated with the proportion of oncologists who were linchpins (medical oncology: ρ, 0.94; 95% CI, 0.93 to 0.95; *P* < .001; radiation oncology: ρ, 0.95; 95% CI, 0.93 to 0.96; *P* < .001). HRRs with more vulnerable networks for medical oncology had a higher percentage of beneficiaries eligible for Medicaid (ρ, 0.19; 95% CI, 0.08 to 0.29; *P* < .001). HRRs with more vulnerable networks for radiation oncology had a higher percentage of beneficiaries living in poverty (ρ, 0.17; 95% CI, 0.06 to 0.27; *P* = .004), a higher percentage of beneficiaries eligible for Medicaid (ρ, 0.21; 95% CI, 0.09 to 0.31; *P* < .001), and lower rates of cohort patients receiving radiation therapy (ρ, –0.18; 95% CI, –0.28 to –0.06; *P* = .003). Oncologist density was inversely associated with poverty and with the proportion of cohort patients receiving chemotherapy; that is, in areas with lower oncologist density, a greater proportion of patients received chemotherapy. Greater oncologist density was associated with more oncologists practicing in nonmetropolitan areas (Table 2).

**Table 2.** Correlations Between HRR Network Vulnerability and Oncologist Density and HRR Sociodemographic Characteristics and Cancer Service Use^a^

Characteristic	ρ (95% CI)			
	Medical Oncology		Radiation oncology	
	Network vulnerability	Oncologist density	Network vulnerability	Oncologist density
% Beneficiaries who are				
Black	0.02 (–0.09 to 0.14)	0.05 (–0.06 to 0.16)	0.09 (–0.03 to 0.20)	–0.02 (–0.13 to 0.10)
Hispanic	0.02 (–0.10 to 0.13)	–0.08 (–0.19 to 0.03)	0.02 (–0.09 to 0.13)	–0.07 (–0.18 to 0.05)
Living in poverty	0.10 (–0.01 to 0.21)	–0.28 (–0.38 to –0.17)^b^	0.17 (0.06 to 0.27)^c^	–0.27 (–0.38 to –0.17)^b^
Eligible for Medicaid	0.19 (0.08 to 0.29)^b^	–0.08 (–0.19 to 0.03)	0.21 (0.09 to 0.31)^b^	–0.12 (–0.23 to –0.01)
Living in metropolitan ZIP codes	0.07 (–0.04 to 0.18)	0.02 (–0.10 to 0.13)	–0.05 (–0.16 to 0.07)	0.02 (–0.09 to 0.13)
Living in micropolitan ZIP codes	–0.11 (–0.22 to –0.00)	0.03 (–0.09 to 0.14)	0.07 (–0.04 to 0.18)	–0.01 (–0.12 to 0.10)
Living in small town ZIP codes	–0.04 (–0.15 to 0.08)	–0.02 (–0.14 to 0.09)	0.05 (–0.07 to 0.16)	–0.05 (–0.16 to 0.07)
Living in isolated ZIP codes	–0.05 (–0.16 to 0.07)	–0.04 (–0.16 to 0.06)	0.01 (–0.10 to 0.13)	–0.01 (–0.11 to 0.11)
% Cohort patients who				
Received radiation therapy	–0.12 (–0.23 to –0.01)	0.05 (–0.07 to 0.16)	–0.18 (–0.28 to –0.06)^c^	0.16 (0.05 to 0.27)^c^
Received chemotherapy	–0.03 (–0.15 to 0.08)	–0.20 (–0.30 to –0.09)^b^	0.04 (–0.07 to 0.15)	–0.22 (–0.32 to –0.11)^b^
% Oncologists who^d^				
Are linchpins^d^	0.94 (0.93 to 0.95)^b^	–0.10 (–0.21 to 0.02)	0.95 (0.93 to 0.96)^b^	–0.18 (–0.29 to –0.06)^c^
Practice in nonmetropolitan ZIP codes	0.07 (–0.05 to 0.18)	0.18^c^ (0.06 to 0.29)	0.09 (–0.03 to 0.20)	0.20 (0.08 to 0.31)^b^

^a^ Values represent Spearman rank correlation coefficient (ρ).

^b^ *P* < .001.

^c^ *P* < .01.

^d^ Calculated for medical and radiation oncologists separately and reported comparisons are for the corresponding network vulnerability and oncologist density.

## Discussion

Using a novel patient-sharing network vulnerability measure based on physician linchpin score, our study suggests that vulnerable networks track with indicators of socioeconomic disadvantage and that network vulnerability was associated with lower rates of radiation therapy. The association between physician network vulnerability and poverty is relevant to recent work demonstrating the role of persistent poverty on cancer health disparities.^11^ Although oncologists practicing in nonmetropolitan ZIP codes were more likely to be linchpins, network vulnerability was not associated with the percentage of Medicare beneficiaries living in rural ZIP codes. This finding is potentially subject to ecological fallacy, whereby interpretations of aggregate findings do not reflect variations among individuals. The extent to which rural individuals receive care from linchpin oncologists and subsequent impacts on outcomes can be examined in future work. Network-based measures of access to care may shed light on conflicting findings on the extent to which rural vs urban-residing patients with cancer experience delayed care and worse outcomes.^12,13^ The high correlation between network vulnerability and the proportion of oncologists who are linchpins implies that the number of beneficiaries in a region induced little association between the number of linchpin oncologists and the total number of oncologists. Finally, we found that HRRs with lower oncologist density had higher rates of chemotherapy use. This may be in part due to the increased risk of late-stage diagnoses in areas with limited oncologists,^14^ but additional information on cancer stage would be needed to test this hypothesis.

### Limitations

The proposed measures of linchpin score and network vulnerability are dependent on the composition of physicians in the underlying patient-sharing network and the threshold used to characterize a physician as a linchpin. In this study, we included all physicians who met our inclusion criteria in the network; however, alternative approaches for network assembly based only on encounters with oncologists or oncologists and general practitioners can be considered. The research question of interest may guide which network is most appropriate. Results from this study are limited to the interpretations of HRR-level analyses, which may not reflect associations at the individual patient level. Additional work examining the network characteristics of physicians providing care to rural and urban patients is needed to fully understand the relationship between patient rurality, linchpin oncologists, and network vulnerability. Extending these analyses to populations beyond fee-for-service Medicare, with data sources, such as state all-payer claims, will broaden generalizability, as networks assembled from Medicare claims data are only a partial representation of cancer care delivery networks.

## Conclusions

The findings of this study suggest that policymakers may consider network vulnerability in addition to oncologist density when making decisions regarding equity of resource allocation and implementation of new cancer care delivery models. Examining the influence of physician linchpin score on care delivery is anticipated to identify strategies by which networks can be leveraged to improve cancer health outcomes by identifying and intervening in the most vulnerable areas.

## Notes

Supplement 1. **eTable 1.** *ICD-10* and CPT codes used to identify cancer patient cohorts **eTable 2. **CPT, *ICD-10*, and HCPCS codes to identify cancer services **eTable 3.** Assessment of a range linchpin score thresholds **eTable 4.** Sociodemographic and socioeconomic characteristics of study cohort patients **eFigure.** Study cohort flow diagram

Supplement 2.
Data Sharing Statement
