# Supplementary material for: Use of a Novel Network-Based Linchpin Score to Characterize Accessibility to the Oncology Physician Workforce in the United States
Source: JAMA Netw Open. 2022 Dec 16;5(12):e2245995. doi: 10.1001/jamanetworkopen.2022.45995 (PMC9856409; doi:10.1001/jamanetworkopen.2022.45995)
Supplement: Supplement 1. — eTable 1. ICD-10 and CPT codes used to identify cancer patient cohorts eTable 2. CPT, ICD-10, and HCPCS codes to identify cancer services eTable 3. Assessment of a range linchpin score thresholds eTable 4. Sociodemographic and socioeconomic characteristics of study cohort patients eFigure. Study cohort flow diagram [file jamanetwopen-e2245995-s001.pdf]

Supplemental Online Content

Moen EL, Brooks GA, O'Malley AJ, Schaefer A, Carlos HA, Onega T. Use of a novel network-based linchpin score to characterize accessibility to the oncology physician workforce in the United States. *JAMA Netw Open.* 2022;5(12):e2245995. doi:10.1001/jamanetworkopen.2022.45995

eTable 1. *ICD-10* and CPT codes used to identify cancer patient cohorts
eTable 2. CPT, *ICD-10*, and HCPCS codes to identify cancer services
eTable 3. Assessment of a range linchpin score thresholds
eTable 4. Sociodemographic and socioeconomic characteristics of study cohort patients
eFigure. Study cohort flow diagram

This supplemental material has been provided by the authors to give readers additional information about their work.

© 2022 Moen EL et al. *JAMA Network Open.*

eTable 1. *ICD-10* and CPT codes used to identify cancer patient cohorts

Diagnoses and Procedures	Codes
Breast cancer diagnosis	ICD-10: C50.x<br>ICD-9: 174.xx, 233.0
Breast cancer biopsy	CPT: 10021, 10022, 19000, 19001, 19081, 19082, 19083, 19084, 19085, 19086, 19100, 19101, 19120, 19125, 19126, 19281, 19282, 19283, 19284, 19285, 19286, 19287, 19288,<br><br>ICD-10: 0HBT0ZX, 0HBT3ZX, 0HBT4ZX, 0HBT7ZX, 0HBT8ZX, 0HBU0ZX, 0HBU3ZX, 0HBU4ZX, 0HBU7ZX, 0HBU8ZX, 0HBV0ZX, 0HBV3ZX, 0HBV4ZX, 0HBV7ZX, 0HBV8ZX
Colorectal cancer diagnosis	ICD-10: C18.x, C19, C20<br>ICD-9: 153.0, 153.1, 153.2, 153.3, 153.4, 153.5, 153.6, 153.7, 153.8, 153.9, 154.0, 154.1, 159.0
Colorectal cancer biopsy	CPT: 45305, 45308, 45309, 45315, 45320, 45331, 45333, 45338, 45342, 45380, 45383, 45384, 45385, 44100<br>The following require same day CPT code 88305: G0121, G0105, 45378, 45379, 45386, 45387, 45391, 45392<br><br>ICD-10: 0DBE0ZX, 0DBE3ZX, 0DBE4ZX, 0DBE7ZX, 0DBE8ZX, 0DBF0ZX, 0DBF3ZX, 0DBF4ZX, 0DBF7ZX, 0DBF8ZX, 0DBG0ZX, 0DBG3ZX, 0DBG4ZX, 0DBG7ZX, 0DBG8ZX, 0DBK0ZX, 0DBK3ZX, 0DBK4ZX, 0DBK7ZX, 0DBK8ZX, 0DBL0ZX, 0DBL3ZX, 0DBL4ZX, 0DBL7ZX, 0DBL8ZX, 0DBM0ZX, 0DBM3ZX, 0DBM4ZX, 0DBM7ZX, 0DBM8ZX, 0DBN0ZX, 0DBN3ZX, 0DBN4ZX, 0DBN7ZX, 0DBN8ZX, 0DBP0ZX, 0DBP3ZX, 0DBP4ZX, 0DBP7ZX, 0DBP8ZX
Lung cancer diagnosis	ICD-10: C34.x<br>ICD-9: 162.xx
Lung cancer biopsy	CPT: 32400, 32402, 32405, 32604, 31623, 31624, 31625, 31628, 31629, 31640, 31632**,** 31633, 32096, 32097, 32098<br><br>ICD-10: 0BBC0ZX, 0BBC3ZX, 0BBC4ZX, 0BBC7ZX, 0BBC8ZX, 0BBD0ZX, 0BBD3ZX, 0BBD4ZX, 0BBD7ZX, 0BBD8ZX, 0BBF0ZX, 0BBF3ZX, 0BBF4ZX, 0BBF7ZX, 0BBF8ZX, 0BBG0ZX, 0BBG3ZX, 0BBG4ZX, 0BBG7ZX, 0BBG8ZX, 0BBH0ZX, 0BBH3ZX, 0BBH4ZX, 0BBH7ZX, 0BBH8ZX, 0BBJ0ZX, 0BBJ3ZX, 0BBJ4ZX, 0BBJ7ZX, 0BBJ8ZX, 0BBK0ZX, 0BBK3ZX, 0BBK4ZX, 0BBK7ZX, 0BBK8ZX, 0BBL0ZX, 0BBL3ZX, 0BBL4ZX, 0BBL7ZX, 0BBL8ZX, 0BBM0ZX, 0BBM3ZX, 0BBM4ZX, 0BBM7ZX, 0BBM8ZX

Note. Cohort patients had a cancer-directed biopsy followed by two ICD-10 cancer diagnosis codes on separate dates within 12 months following biopsy. Patients with an ICD-9 or ICD-10 cancer diagnosis code in the 12 months preceding the biopsy were excluded.

© 2022 Moen EL et al. *JAMA Network Open.*

eTable 2. CPT, *ICD-10*, and HCPCS codes to identify cancer services

Service	Codes
Radiation	**CPT:** 77261, 77262, 77263, 77280, 77281, 77282, 77283, 77284 77285, 77286, 77287, 77289 77290,<br><br>77295, 77300, 77301, 77331, 77338, 77306, 77307, 77316, 77317, 77318, 77321, 77332, 77333, 77334, 77336, 77370.<br><br>77761, 77762, 77763, 77767, 77768, 77770, 77771, 77772, 77778, 0394T, 0395T, 77789, 77750, 77790, 77401, 77402, 77403, 77404, 77405, 77406, 77407, 77408, 77409, 77410, 77411, 77412, 77413, 77414, 77415, 77416, 77385, 77386, 77424, 77425, 77422, 77423, 77520, 77521, 77522, 77523, 77524, 77525, 77371, 77372, 77373, 77387, 77014, 77427, 77431, 77432, 77435, 77469, 77470<br><br>**ICD-10**<br>Lung and bronchus: DB01.XX and DB02.XX<br>Breast: DM00.XX, DM01.XX<br>Colon and rectum: DD05.XX, DD07.XX, DD15.XX, DD17.XX<br><br>**REV Code**<br>0330, 0333
Chemotherapy	**CPT:** 36640, 51720, 96400, 96405, 96406, 96408, 96410, 96412, 96414, 96420, 96422, 96423, 96425, 96440, 96445, 96450, 96501, 96504, 96505, 96508, 96510, 96511, 96512, 96520, 96524, 96530, 96538, 96540, 96542, 96545, 96549, 96450, 96555<br><br>**HCPCS**: J0202, J0594, J0894, J1675, J1930, J1950, J2353, J2354, J2860, J3315, J3316, J7504, J7511, J8510, J8520, J8530, J8560, J85662, J8565, J8600, J8700, J8705, J8999, J9000-J9999;<br><br>**REV Code**<br>0331, 0332, 0335

© 2022 Moen EL et al. *JAMA Network Open.*

eTable 3. Assessment of a range linchpin score thresholds

Linchpin score threshold	Linchpin medical oncologists N (% of 7221[a])	Linchpin radiation oncologists N (% of 3573[b])	Total number of linchpins per HRR Median (IQR)
0.1	1096 (15.2)	885 (24.8%)	4 (2, 8)
0.2	781 (10.8)	690 (19.3)	3 (1, 6)
0.3	541 (7.5)	551 (15.4)	2 (1, 4)
0.4	416 (5.8)	423 (11.8)	1 (0, 3)
0.5	298 (4.1)	298 (8.3)	1 (0, 2)

[a]total number of medical oncologists; [b]total number of radiation oncologists

© 2022 Moen EL et al. *JAMA Network Open.*

eTable 4. Sociodemographic and socioeconomic characteristics of study cohort patients.

		Breast cancer		Colorectal cancer		Lung cancer	
		N = 161,206		N = 76,604		N = 70,904	
Sex		N	%	N	%	N	%
	Male	0	0.0%	37,364	48.8	33,025	46.6%
	Female	161,206	100.0	39,240	51.2	37,879	53.4%
Age in years							
	65 to 69	41,882	26.0	15,689	20.5%	14,580	20.6%
	70 to 74	47,903	29.7	19,596	25.6%	20,915	29.5%
	75 to 79	34,373	21.3	17,567	22.9%	17,841	25.2%
	80 to 84	20,820	12.9	13,084	17.1%	11,217	15.8%
	85 to 89	11,372	7.1	7,782	10.2%	5,235	7.4%
	90 to 94	4,078	2.5	2,570	3.4%	1,044	1.5%
	95 to 99	778	0.5	316	0.4%	72	0.1%
Race/Ethnicity							
	Asian	1,953	1.2	1,136	1.5%	795	1.1%
	Black	11,555	7.2	5,662	7.4%	4,391	6.2%
	Hispanic	1,134	0.7	733	1.0%	386	0.5%
	North American Native	558	0.3	399	0.5%	361	0.5%
	Other	2,175	1.3	1,088	1.4%	740	1.0%
	Unknown	1,669	1.0	910	1.2%	644	0.9%
	White	142,162	88.2	66,676	87.0%	63,587	89.7%
Rurality							
	Metropolitan	127,029	78.8	57,896	75.6%	53,678	75.7%
	Micropolitan	17,975	11.2	9,705	12.7%	8,986	12.7%
	Small town	9,442	5.9	5,328	7.0%	4,881	6.9%
	Isolated	6,751	4.2	3,673	4.8%	3,359	4.7%
Percent Poor 65+, mean and SD		8.40	5.40	8.90	5.70	8.70	5.50
Received chemotherapy		34,339	21.3	20,044	26.2%	29,971	42.3%
Received radiation therapy		86,064	53.4	11,365	14.8%	36,506	51.5%

SD = standard deviation

© 2022 Moen EL et al. *JAMA Network Open.*

eFigure 1. Study cohort flow diagram.

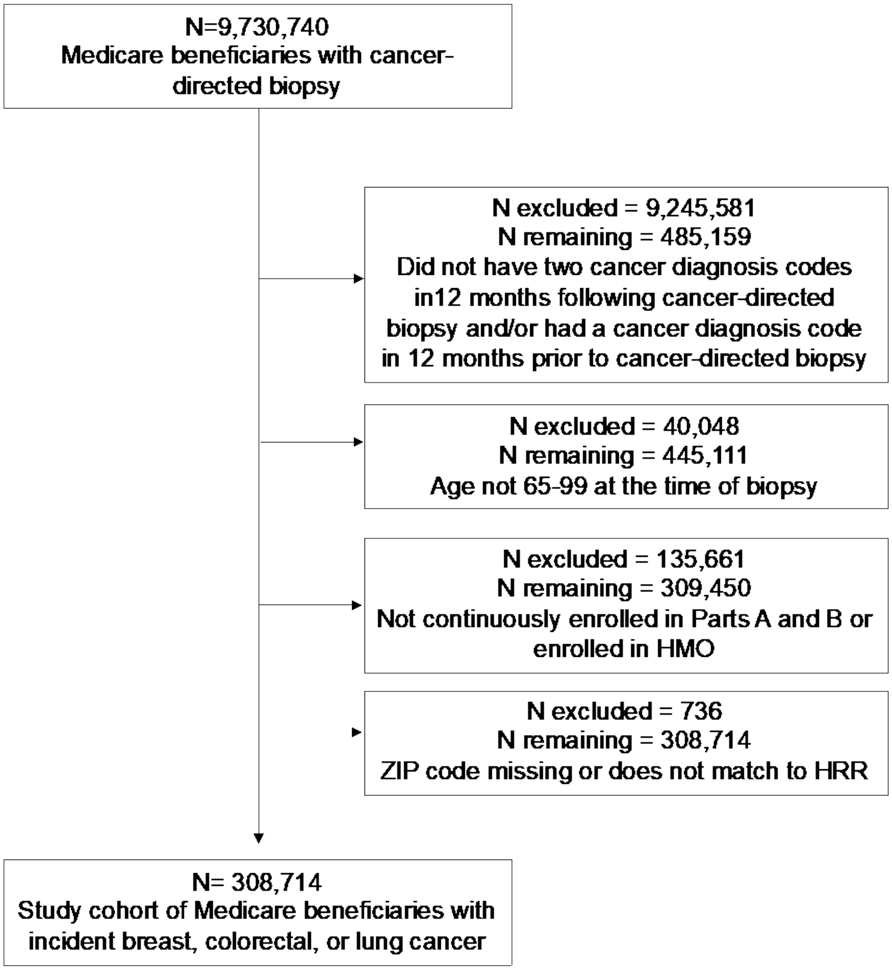

© 2022 Moen EL et al. *JAMA Network Open.*
