# Supplementary material for: Use of a Novel Network-Based Linchpin Score to Characterize Accessibility to the Oncology Physician Workforce in the United States
Source: JAMA Netw Open. 2022 Dec 16;5(12):e2245995. doi: 10.1001/jamanetworkopen.2022.45995 (PMC9856409; doi:10.1001/jamanetworkopen.2022.45995)
Supplement: Supplement 2. — Data Sharing Statement [file jamanetwopen-e2245995-s002.pdf]

Data Sharing Statement

Moen. Use of a Novel Network-Based Linchpin Score to Characterize Accessibility to the Oncology Physician Workforce in the United States. *JAMA Netw Open*. Published December 16, 2022. doi:10.1001/jamanetworkopen.2022.45995

Data
Data available: No

Additional Information
Explanation for why data not available: In accordance with the Data Use Agreement with CMS, the authors are not at liberty to share patient-level data with other parties.
